# Risk factors of frailty and death or only frailty after intensive care in non-frail elderly patients: a prospective non-interventional study

**DOI:** 10.1186/s40560-019-0403-3

**Published:** 2019-10-30

**Authors:** Yoann Launey, Hervé Jacquet, Matthieu Arnouat, Chloe Rousseau, Nicolas Nesseler, Philippe Seguin

**Affiliations:** 1 CHU Rennes, Université Rennes 1, Service de Réanimation Chirurgicale, Hôpital Pontchaillou, 2 rue Henri Le Guilloux, 35033 Rennes Cedex 9, France; 2 CHU de Rennes, Centre d’Investigation Clinique, Inserm 1414, 2 rue Henri Le Guilloux, 35033 Rennes Cedex 9, France; 3 Inserm, Institut NUMECAN – UMR_A 1341, UMR_S 1241, F-35000 Rennes, France; 4 Service d’Anesthésie Réanimation 1, Hôpital de Pontchaillou, 2 rue Henri Le Guilloux, 35033 Rennes Cedex 9, France

**Keywords:** Critical care, Non-frail, Elderly, Outcome

## Abstract

**Background:**

Frailty status is recognized as an important parameter in critically ill elderly patients, but nothing is known about outcomes in non-frail patients regarding the development of frailty or frailty and death after intensive care. The aim of this study was to determine risk factors for frailty and death or only frailty 6 months after intensive care unit (ICU) admission in non-frail patients ≥ 65 years.

**Methods:**

A prospective non-interventional study performed in an academic ICU from February 2015 to February 2016 included non-frail ≥ 65-year-old patients hospitalized for > 24 h in the ICU. Frailty was assessed by calculating the frailty index (FI) at admission and 6 months later. Patients who remained non-frail (FI < 0.2) were compared to patients who presented frailty (FI ≥ 0.2) and those who presented frailty and death at 6 months.

**Results:**

Among 974 admissions, 136 patients were eligible for the study and 88 patients were analysed at 6 months (non-frail *n* = 34, frail *n* = 29, death *n* = 25). Multivariable analysis showed that mechanical ventilation duration was an independent risk factor for frailty/death at 6 months (per day of mechanical ventilation, odds ratio [OR] = 1.11; 95% confidence interval [CI] 1.04–1.19, *p* = 0.002). When excluding patients who died, mechanical ventilation duration remained the sole risk factor for frailty at 6 months (OR = 1.19; 95% CI 1.07–1.33, *p* = 0.001).

**Conclusion:**

Mechanical ventilation duration was the sole predictive factor of frailty and death or only frailty 6 months after ICU hospitalization in initially non-frail patients.

## Introduction

As life expectancy increases, the need for hospitalization in the general ward and the intensive care of elderly patients is increasing, representing a major challenge over the next few years in an increasingly financially constrained system [1–4]. Faced with this demographic challenge, it is now well accepted that age itself is not relevant for intensive care unit (ICU) admission decisions and that other parameters must be taken into account, notably, comorbidities and functional status [5]. Frailty, a geriatric concept developed at the end of the last century in the USA and Canada, is defined as a decrease in physiological reserves inducing an alteration in the mechanisms of adaptation to stress [6]. There is now clear evidence that frail elderly patients hospitalized in ICUs are at particular risk of early complications, long-term disabilities and death, independent of age and usual severity markers [7–12]. Additionally, some patients considered non-frail at admission will become frail or will die after ICU hospitalization. To the best of our knowledge, no study has evaluated the factors that contribute to a non-frail patient at admission becoming frail and dying or becoming frail after ICU hospitalization.

In this context, the objective of this study was to determine the risk factors for becoming frail and dying or becoming frail 6 months after ICU hospitalization in initially non-frail patients older than 65 years.

## Patients and methods

This was a prospective, non-interventional study conducted from February 2015 to February 2016 in the ICU of the University Hospital of Rennes. Within this period, all patients aged 65 years or older admitted to intensive care for > 24 h and whose frailty index (FI) was < 0.2 were eligible. Patients who were unable to answer questions, under curatorship and/or for whom no relatives were present or able to answer questions were excluded. During the period of study, only the first admission was considered. The protocol was approved by the Ethics Committee of the University Hospital of Rennes. Institutional review board waived written informed consent according to the no-interventional study design.

The main objective was to compare the initially non-frail patients admitted to the ICU who remained non-frail at 6 months with those who developed frailty and/or died at 6 months. In addition, the probability of developing moderate or severe frailty or dying was assessed.

### Frailty assessment

Frailty was assessed by measuring the FI [13]. This measurement was based on an interview with the patient (or relatives if the patient was unable to answer questions) using a previously validated 40-item questionnaire; 6 items (mini-mental state examination, grip strength, shoulder strength, peak flow, usual pace and walking pace) were removed because they could not be measured in ICU patients, and heart attack was associated with chronic heart failure [13]. Accordingly, a total of 33 items, including different domains related to health status, were explored (see Additional file 1), and questions focused on autonomy for activities of daily living, physical and emotional health, the presence of chronic condition(s), cognition, nutritional status and objective respiratory parameters (Additional file 1). Each item was assigned a point value ranging from 0 (not at all altered) to 1 (completely altered). The FI was obtained by dividing the number of points by the number of items evaluated. For example, if the total points were 10 out of 30 items for which a response could be obtained, the FI was 0.33 (10/30). This score ranged from 0 to 1, and frailty was defined as an FI ≥ 0.2 [13]. A response for at least 30 variables was recommended for an FI calculation [13]. The interview was conducted during the first 72 h of ICU hospitalization by 2 ICU physicians. Only patients with an FI < 0.2 at admission were included. The FI was measured again at 6 months, and patients who remained non-frail (FI < 0.2) were compared to those who developed frailty and died, and those who developed only frailty. At 6 months, frailty was assessed by phone by the same 2 ICU physicians using the same questionnaire administered at admission.

### Data collection

The following data were collected: age, sex, body mass index, marital status, usual place of residence, ICU hospitalization in the 6 months preceding the current hospitalization and reason for admission (medical, trauma or unscheduled or scheduled surgery). Severity was assessed by the severity acute physiologic assessment II (SAPS II) and by the Sequential Organ Failure Assessment (SOFA) scores [14, 15]. Life expectancy was estimated by the McCabe score [16]. The activity of daily living was assessed by the Katz index and comorbidities were assessed by the Charlson index [17, 18]. The following clinical data at admission were also noted: shock and, if present, the type of shock; infection and, if present, the site of infection; and the severity of infection (sepsis or septic shock) and bacteraemia.

During the entire ICU hospitalization period, the following data were also collected: use of vasopressor and the maximal dose, the presence of acute respiratory distress syndrome according to the Berlin definition [19], the occurrence of acute renal failure according to the risk, injury, failure, loss, and end-stage renal failure (RIFLE) score [20]; the maximum level of plasmatic creatinine observed; the use of extra-renal replacement therapy; and the need and duration of mechanical ventilation. Sedation and its duration were also noted, as well as the mean doses of sedative agents (mg/h) and the use of neuro-muscular blocking agents. The administration of enteral nutrition and the mean Kcal/kg day^−1^ for days 0 to 5 and 6 to 10 were also recorded as well as the dose of protein administered in g/kg day^−1^ for days 0 to 5 and 6 to 10. The acquisition of infection during ICU hospitalization, its severity and the number of acquired infections were noted. Finally, mobilization and out of bed were notified and reported as at least one action once during ICU stay.

The ICU and hospital length of stay, the decision to withdraw or withhold life-sustaining therapies during the ICU stay and mortality were reported.

### Statistical analyses

The statistical analyses were performed using SAS software version 9.4 (SAS Institute, Cary, NC). The quantitative variables were reported as the median (interquartile range 25–75) and compared by non-parametric Mann-Whitney Wilcoxon tests, and the qualitative variables were reported as *n* (percentage) and compared by *χ*^2^ or non-parametric Fisher’s tests as required. Patients who remained non-frail at 6 months were compared to patients who developed frailty and died, and the analysis was extended to patients who survived but developed frailty at 6 months. A *p* value < 0.05 was considered statistically significant. The risk factors for frailty and death and only frailty at 6 months were then tested by logistic regression. Significantly clinically relevant parameters in univariate analysis at *p* < 0.20 were included in a multivariate model, and a stepwise top-down selection was performed.

## Results

**Fig. 1:**
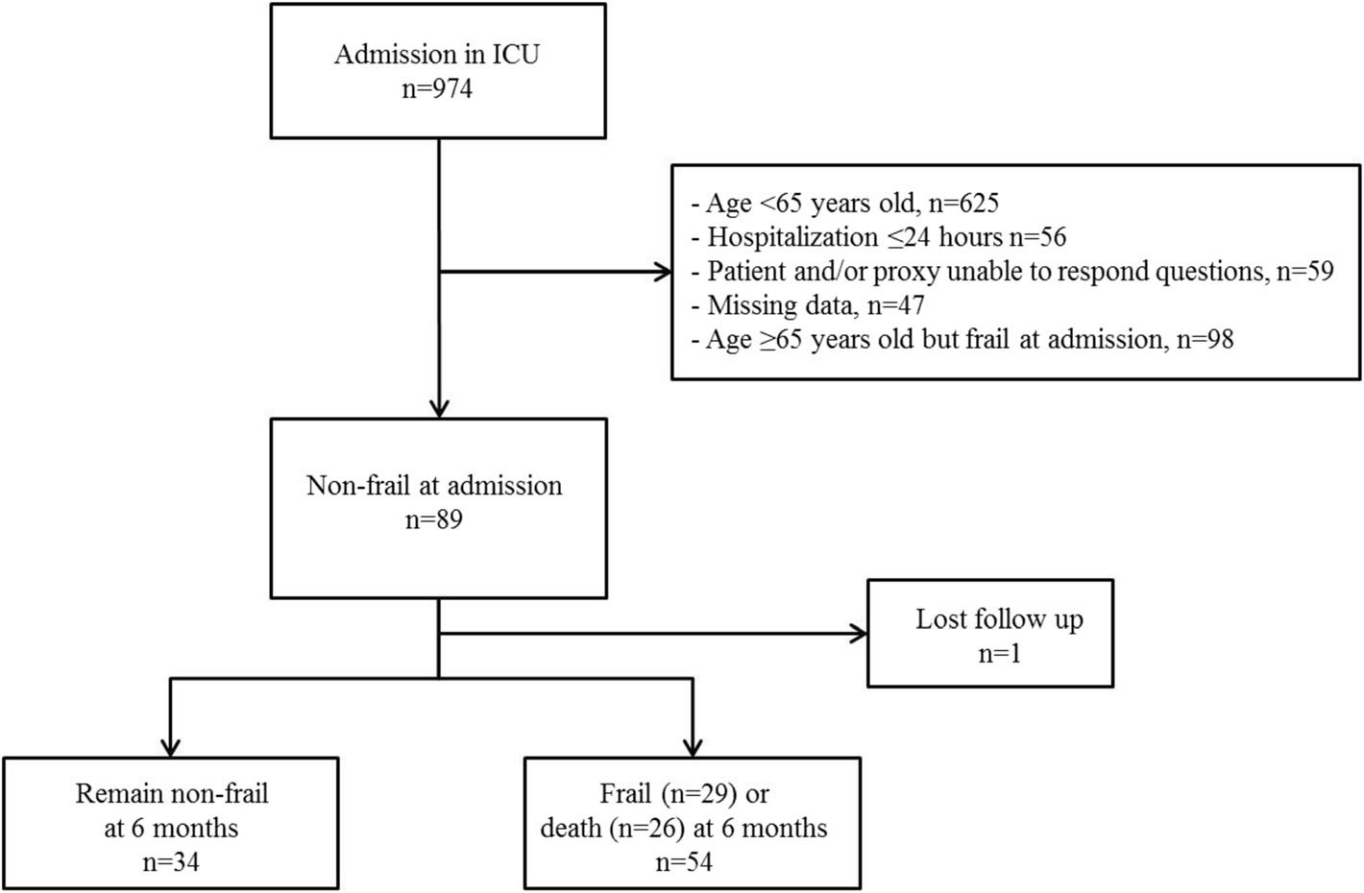
Flow chart

During the study period, a total of 974 patients were admitted to the ICU and 89 patients ≥ 65 years old were non-frail at admission (FI < 0.2) (Fig. 1). One patient was lost before follow-up at 6 months and was therefore excluded from the analysis. Accordingly, a total of 88 patients were analysed: 34 remained non-frail at 6 months and 54 developed frailty or died (frail *n* = 29 and dead *n* = 25) at 6 months.

When we compared patients who remained non-frail at 6 months to those who developed frailty or died at 6 months, the baseline characteristics and clinical data at admission of the two groups were comparable; however, patients who developed frailty or died at 6 months were significantly more likely to have been hospitalized in the 6 months prior to inclusion (Tables 1 and 2). Clinical data recorded during the entire ICU hospitalization and data related to the occurrence of infection during ICU hospitalization but not present at admission are reported in Tables 3 and 4, respectively. The durations of mechanical ventilation and sedation were significantly higher in patients who developed frailty or died, and patients who remained non-frail received significantly less Kcal/kg day^−1^ and less protein (g/kg d^−1^) via enteral nutrition during the first 5 days of hospitalization. The ICU and hospital length of stay, withdrawal or withholding of life-sustaining therapies in the ICU and the ICU, hospital and 6-month mortalities are presented in Table 5. The ICU and hospital lengths of stay were significantly longer in the group of patients who developed frailty or died at 6 months. The following variables were included in the multivariable analysis model: age, hospitalization during the 6 months preceding the current hospitalization, the Charlson index, infection during the current ICU stay, mechanical ventilation duration, mean daily dose of enteral nutrition between days 0 and 5 and hospital length of stay. After stepwise downward selection, only the duration of mechanical ventilation was an independent risk factor for progression to frailty/death at 6 months (per day of mechanical ventilation, odds ratio [OR] = 1.11; 95% confidence interval [CI] 1.04–1.20, *p* = 0.002).

**Table 1:** Baseline characteristics of patients remaining non-frail vs those who developed frailty and died and those who developed only frailty

	Non-frail at 6 months (*n* = 34)	Frail or death at 6 months (*n* = 54)	P*	Frail at 6 months (*n* = 29)	P**
Age, years	71 (66–77)	75 (68–81)	0.102	73 (68–77)	0.403
Sex, male	22 (65)	33 (61)	0.734	19 (66)	0.946
Body mass index, Kg/m^2^	28 (25–29)	27 (24–28)	0.278	27 (24–30)	0.267
Married or common law	23 (82)	31 (70)	0.264	31 (69)	0.210
Residence			1.000	29 (100)	1.000
Living at home	34 (100)	53 (98)		0 (−)	–
Nursing home	0 (−)	1 (2)			
Previous (6 months) ICU stay	2 (6)	15 (28)	0.011	8 (28)	0.035
Type of admission			0.321		0.559
Unscheduled surgery	12 (35)	25 (46)		14 (48)	
Scheduled surgery	7 (21)	10 (18)		5 (17)	
Medical	9 (26)	16 (30)		8 (28)	
Trauma	6 (18)	3 (6)		2 (7)	
SAPS II	43 (34–54)	44 (36–54)	0.693	39 (35–50)	0.396
SOFA	6 (3–9)	5 (4–9)	0.711	4 (3–7)	0.176
Mac Cabe			0.455		1.00
A	25 (73)	32 (59)		21 (73)	
B	7 (21)	16 (30)		7 (24)	
C	2 (6)	6 (11)		1 (3)	
Frailty index at admission, mean ± SD	0.08 ± 0.06	0.10 ± 0.05	0.074	0.10 ± 0.06	0.119
Katz score	6 (6–6)	6 (6–6)	1.000	6 (6–6)	–
Charlson score	1 (0–2)	1 (0–2)	0.121	1 (0–2)	0.466

Data are expressed as median (interquartile range 25–75) and number (percentage) or otherwise mentioned. *Non-frail vs frail or died at 6 months. **Non-frail vs frail at 6 months. *SAPS II* severity acute physiologic assessment II, *SOFA* Sequential Organ Failure Assessment

**Table 2:** Clinical data at admission for patients remaining non-frail vs those who developed frailty and died and those who developed only frailty

	Non-frail at 6 months (*n* = 34)	Frail or death at 6 months (*n* = 54)	P*	Frail at 6 months (*n* = 29)	P**
Shock	15 (44)	23 (43)	0.888	11 (38)	0.619
Type of shock			0.076		0.072
Septic	9 (60)	10 (48)		5 (45)	
Cardiogenic	3 (20)	1 (4)		0 (−)	
Hypovolemic	2 (13)	10 (44)		6 (54)	
Haemorrhagic	0 (−)	1 (4)		0 (−)	
Others	1 (7)	0 (−)		0 (−)	
Infection	12 (35)	18 (35)	0.850	9 (31)	0.721
Site of infection			0.871		0.661
Abdominal	4 (33)	5 (28)		1 (11)	
Urinary	2 (17)	3 (17)		2 (22)	
Pulmonary	1 (8)	4 (22)		2 (22)	
Mediastinal	2 (17)	4 (22)		3 (33)	
Others	3 (25)	2 (11)		1 (11)	
Bacteraemia	3 (27)	2 (11)	0.339	1 (11)	0.591
Infection severity			0.645		0.676
Sepsis	1 (8)	3 (17)		2 (22)	
Severe sepsis	2 (17)	5 (28)		2 (22)	
Septic shock	9 (75)	10 (55)		5 (56)	

Data are expressed as number (percentage). *Non-frail vs frail or died at 6 months. **Non-frail vs frail at 6 months

**Table 3:** Infection occurring during ICU hospitalization not present at admission

	Non-frail at 6 months (*n* = 34)	Frail or death at 6 months (*n* = 54)	P*	Frail at 6 months (*n* = 29)	P**
Infection, yes	4 (12)	16 (30)	0.051	7 (24)	0.197
Infection severity			0.249		0.524
Sepsis	3 (75)	3 (23)		3 (43)	
Severe sepsis	0 (−)	3 (23)		0 (−)	
Septic shock	1 (25)	7 (54)		4 (57)	

Data are expressed as number (percentage). *Non-frail vs frail or died at 6 months. **Non-frail vs frail at 6 months

**Table 4:** Clinical data during the entire hospitalization in ICU

	Non-frail at 6 months (*n* = 34)	Frail or death at 6 months (*n* = 54)	P*	Frail at 6 months (*n* = 29)	P**
Norepinephrine, yes	17 (49)	34 (63)	0.230	17 (59)	0.494
Norepinephrine, max dose, μg/kg min^−1^	0.3 (0.2–0.6)	0.5 (0.3–0.7)	0.123	0.4 (0.3–0.5)	0.500
ARDS			0.698		0.594
No	30 (88)	47 (87)		26 (90)	
Moderate	3 (9)	3 (6)		1 (3)	
Severe	1 (3)	4 (7)		2 (7)	
Creatinin max, μmol l^−1^	106 (71–172)	95 (70–169)	0.687	75 (66–105)	0.121
Score RIFLE			0.902		0.532
No renal aggression	12 (35)	21 (39)		15 (52)	
“Risk”	7 (21)	11 (20)		6 (21)	
“Injury”	7 (21)	7 (13)		3 (10)	
“Failure”	8 (23)	14 (26)		5 (17)	
“Loss”	0 (−)	1 (2)		0 (−)	
Extra renal support	6 (18)	13 (24)	0.475	4 (14))	0.741
Mechanical ventilation	28 (82)	43 (79)	0.753	25 (86)	0.741
Length of mechanical ventilation, days	2 (1–5)	10 (3–21)	0.002	5 (3–17)	0.006
Sedation use	23 (68)	40 (74)	0.515	21 (72)	0.681
Length of sedation, days	1 (1–3)	3 (2–6)	0.002	1 (1–4)	0.095
Mean dose of midazolam, mg h^−1^	1.2 (1.0–1.5)	1.3 (0.9–1.6)	0.793	1.2 (0.9–1.5)	0.902
Mean dose of morphine, mg h^−1^	1.9 (1.0–2.9)	2.7 (1.5–3.8)	0.178	2.1 (1.2–4.3)	0.488
Use of neuro-blocking agent	3 (9)	9 (17)	0.356	3 (10)	1.000
Rehabilitation^†^					
Mobilization	19 (56)	40 (74)	0.125	20 (69)	0.420
Out of bed	15 (44)	31 (57)	0.319	19 (65)	0.148
Enteral nutrition	26 (77)	46 (85)	0.302	26 (90)	0.170
Median daily dose of enteral nutrition delivered, Kcal/kg day^−1^					
Between day 0 and day 5	8 (5–12)	11 (7–15)	0.050	11 (6–16)	0.151
Between day 6 and day 10	16 (11–22)	15 (7–21)	0.547	15 (6–22)	0.599
Median daily protein delivered via enteral nutrition, g/kg day^−1^					
Between day 0 and day 5	0.3 (0.2–0.5)	0.5 (0.3–0.6)	0.025	0.5 (0.2–0.6)	0.064
Between day 6 and day 10	0.7 (0.5–0.8)	0.7 (0.4–0.8)	0.532	0.6 (0.5–0.9)	0.463

Data are expressed as median (interquartile range 25–75) or number (percentage). *Non-frail vs frail or died at 6 months. **Non-frail vs frail at 6 months. ^†^Mobilization and out of bed was notified as at least one action once during ICU stay. *ARDS* Acute respiratory distress syndrome, *RIFLE* risk, injury, failure, loss, end-stage renal disease, *ICU* intensive care unit

**Table 5:** Length of stay, decision of withdrawal or withholding of life-sustaining therapies in ICU and mortality

	Robust at 6 months (*n* = 34)	Frail or dead at 6 months (*n* = 54)	P*	Frail at 6 months (*n* = 29)	P**
Length of stay, days					
ICU	5 (4–9)	15 (7–27)	< 0.001	15 (7–24)	< 0.001
Hospital	28 (12–40)	45 (21–63)	0.003	48 (29–63)	< 0.001
WWLST* in ICU	0 (−)	17 (31)		2 (7)	
Mortality					
ICU	–	13 (24)		–	
Hospital	–	18 (33)		–	
At 6 months	–	25 (46)		–	

Data are expressed as median (interquartile range 25–75) or number (percentage). *Non-frail vs frail or died at 6 months. **Non-frail vs frail at 6 months. *ICU* intensive care unit, *WWLST* withdrawal or withholding of life-sustaining therapies

When we excluded patients who died and compared patients who remained non-frail with those who developed frailty at 6 months, the univariate analysis revealed the same data to be significant, except for the duration of sedation, which did not differ between the two groups (Tables 1, 2, 3, 4 and 5). The following variables were included in the multivariate analysis model: SOFA, ICU stay in the 6 months preceding the current hospitalization, duration of mechanical ventilation, mean daily enteral nutrition between days 0 and 5 and hospital length of stay. Similarly, only the duration of mechanical ventilation was found to be a risk factor for frailty at 6 months (per day of mechanical ventilation, OR = 1.19; 95% CI 1.07–1.33, *p* = 0.001).

The details of the items included in the questionnaire are provided in Additional file 2. In patients who were non-frail at admission but developed frailty at 6 months, the primary difficulties reported by the patients at 6 months were related to basic and instrumental daily living activities, mobility, strength and changes in health status.

## Discussion

To the best of our knowledge, this prospective non-interventional study is the first to investigate the risk factors for developing frailty and dying or developing only frailty 6 months after ICU hospitalization in elderly patients initially considered non-frail at admission. It appeared that the initial clinical data, notably, usual severity factors (SAPS II, SOFA), did not differ between the groups, suggesting that the occurrence of event(s) during the ICU stay rather than existing elements at admission were “tipping” factors in progressing from a non-frail to a frail status or death. In the multivariable analysis, only the duration of mechanical ventilation was an independent predictor of progression to frailty and death or only frailty in initially non-frail patients. Accordingly, it may be hypothesized that mechanical ventilation affects such a progression, but the mechanism for this progression needs further discussion.

From this perspective, hypomobility may be an important parameter to consider. Indeed, it has been shown that patients under mechanical ventilation are more prone to bed rest and less mobility. In a single-day study conducted in 38 Australian and New Zealand ICUs, out-of-bed activity was never practised in mechanically ventilated patients [21]. In a 1-day prevalence study performed in 116 ICUs in Germany with 783 mechanically ventilated patients, out-of-bed mobilization was reported in 24% of patients and only 4% could stand, march or walk [22]. In Switzerland, a 1-day prevalence study conducted in 35 ICUs showed that 33% (53/191) of mechanically ventilated patients practised active mobilization, and walking was achieved in only 2% of patients [23]. In a 2-day cross-sectional point prevalence study performed in 42 ICUs with patients who had acute respiratory failure requiring mechanical ventilation > 48 h at any point during their ICU stay, out-of-bed mobility was achieved on 16% of the total patient-days in mechanically ventilated patients and walking was achieved in only 4% of patients [24]. In contrast, non-mechanically ventilated patients spent 46% of patient-days out of bed. In this study, mechanical ventilation through the endotracheal tube or a tracheostomy and delirium were negatively associated with out-of-bed mobility [24]. The impact of bed rest and hypomobility on muscle loss is well described, and elderly people are more prone to bed rest than younger people [25, 26]. For 12 healthy old people remaining in bed for 10 days, it was shown that there was a significant decrease in muscle protein synthesis, whole-body lean mass and strength [25]. Moreover, after 14 days of bed rest, the muscle mass and function were significantly more altered in elderly people (age 55–65 years) than in younger people (age 18–30 years), and the application of a rehabilitation protocol after bed rest did not allow the recovery of the pre-bed rest conditions [26]. In the ICU, the bed rest duration was found to be an independent risk factor for muscle weakness in surviving acute respiratory distress syndrome (ARDS) patients [27, 28]. Clearly, ICU-acquired weakness is multifactorial and not exclusively related to bed rest [29]. Nevertheless, in our study, we did not find a difference between groups for the usual factors of ICU-acquired weakness, i.e. age and sepsis (at admission and during hospital stay); only the duration of mechanical ventilation was independently associated with subsequent frailty or frailty and death. At 6 months, we found that patients who developed frailty had, in comparison with non-frail patients, strong deficits in mobility and strength and had more disabilities in daily living activities and instrumental activities that indirectly reflected the capacity to move (Additional file 2: Figure S1 and S2). The impact of hypomobility on functional capacity was explored in a randomized study comparing standard care to early physical/occupational therapy in patients mechanically ventilated for less than 72 h. In this study, patients in the interventional group were able to walk a greater distance at hospital discharge than the control group and were more independent in daily living activities [30]. Nevertheless, the impact of physical/occupational therapy on outcomes in ICU patients remains controversial, and no study has specifically addressed this issue in elderly ICU patients. A recent meta-analysis found that active mobilization and rehabilitation improved muscle strength and the probability of mobilization without assistance at hospital discharge but did not decrease ICU stay, hospital stay and 6-month mortality [31]. Another critical issue is the type of sedatives/opioids used for sedation/analgesia. Indeed, it has been shown that non-benzodiazepine-based sedation (dexmedetomidine or propofol) may reduce the length of ICU stay and duration of mechanical ventilation [32]. In the same way, remifentanil, a short half-life opioid, appeared to decrease the duration of mechanical ventilation, time to extubation and length of ICU stay [33]. Nevertheless, the impact of half-life sedatives and/or opioids does not appear to have any impact on mortality [32, 33]. In our institution, we used midazolam and morphine as sedative and analgesic agents. This point may have an impact on mechanical ventilation duration, but all our patients received the same agents according to a protocol. Moreover, the doses we used were low and did not differ between the groups.

Several limitations of our study need to be noted. First, it was monocentric, and our results may be difficult to generalize to other ICUs. Second, we chose to evaluate frailty by calculating an FI rather than using the clinical frailty scale (CFS), which is the most useful and widely used tool to evaluate frailty in the ICU [11]. Nevertheless, we used a validated frailty determination method that allowed us to explore and quantify each frailty domain and follow patients for 6 months, which would not be possible with the CFS. Third, we cannot exclude that patients had previous sarcopenia, which may have worsened muscle loss during ICU hospitalization. Indeed, it has been shown that sarcopenia at admission in older trauma patients, evaluated by the area of skeletal muscle on CT scan at the third lumbar vertebra, was associated with increased mortality (26% vs 14%, *p* = 0.008) and independently associated with fewer ventilator-free days and ICU-free days [34]. Accordingly, our patients were more frequently hospitalized within the 6 months prior to ICU admission, possibly favouring some degree of muscle loss, but this variable was not included in the multivariable analysis. Moreover, answers to items of the FI questionnaire specifically oriented to the evaluation of mobility and strength at admission were not altered in patients who remained non-frail and those who developed frailty (Additional file 2: Figure S1 and S2). Finally, we did not study precisely the level and lead time of physical/occupational therapy of patients during their ICU stay, which may differ between groups, but only if they had been mobilized and/or put out of bed at least once during their ICU stay.

## Conclusion

Mechanical ventilation duration appeared to be a strong predictive factor of frailty and death or frailty alone 6 months after ICU hospitalization in patients who were non-frail at admission. Further studies should focus on the sequential evaluation of muscle loss in elderly patients in the ICU and evaluate the effect of early mobilization on the subsequent development of frailty in non-frail patients.

## Supplementary information

**Additional file 1: Table S1**. Details of the questionnaire for the calculation of the frailty index [from Searle SD, Mitnitski A, Gahbauer EA, Gill TM, Rockwood K. A standard procedure for creating a frailty index. BMC Geriatr. 2008;30:24].

**Additional file 2 **Details of the variables from the frailty index, reported at admission (remaining non-frail and becoming frail at 6 months) and at 6 months (patients remaining non-frail and becoming frail). **Figure S1**. Basic and instrumental daily living activities. **Figure S2**. Problems related to mobility and strength. **Figure S3.** Various feelings declared by the patients and trouble getting going. **Figure S4**. Weight loss, altered health status and problems with usual activities. **Figure S5**. Comorbidities.

## Data Availability

Please contact the authors for data requests.

## Abbreviations

ARDS: Acute respiratory distress syndrome
CFS: Clinical frailty scale
FI: Frailty index
ICU: Intensive care unit
RIFLE: Risk, injury, failure, loss, and end-stage renal failure
SAPS II: Severity acute physiologic assessment II
SOFA: Sequential Organ Failure Assessment
