# Supplementary material for: Risk factors of frailty and death or only frailty after intensive care in non-frail elderly patients: a prospective non-interventional study
Source: J Intensive Care. 2019 Oct 30;7:48. doi: 10.1186/s40560-019-0403-3 (PMC6820956; doi:10.1186/s40560-019-0403-3)
Supplement: Supplementary file 1 — Additional file 1: Table S1. Details of the questionnaire for the calculation of the frailty index [from Searle SD, Mitnitski A, Gahbauer EA, Gill TM, Rockwood K. A standard procedure for creating a frailty index. BMC Geriatr. 2008;30:24]. [file 40560_2019_403_MOESM1_ESM.docx]

**Additional file 1**

**Table:** Detail of the questionnaire for the calculation of the frailty index [from Searle SD, Mitnitski A, Gahbauer EA, Gill TM, Rockwood K. A standard procedure for creating a frailty index. BMC Geriatr. 2008; 30: 24].

|  |  |  |  |  |  |
| --- | --- | --- | --- | --- | --- |
| Help Bathing | Yes = 1 | No = 0 |  |  |  |
| Help Dressing | Yes = 1 | No = 0 |  |  |  |
| Help getting in/out of Chair | Yes = 1 | No = 0 |  |  |  |
| Help Walking around house | Yes = 1 | No = 0 |  |  |  |
| Help Eating | Yes = 1 | No = 0 |  |  |  |
| Help Grooming | Yes = 1 | No = 0 |  |  |  |
| Help Using Toilet | Yes = 1 | No = 0 |  |  |  |
| Help up/down Stairs | Yes = 1 | No = 0 |  |  |  |
| Help lifting 10 lbs | Yes = 1 | No = 0 |  |  |  |
| Help Shopping | Yes = 1 | No = 0 |  |  |  |
| Help with Housework | Yes = 1 | No = 0 |  |  |  |
| Help with meal Preparations | Yes = 1 | No = 0 |  |  |  |
| Help taking Medication | Yes = 1 | No = 0 |  |  |  |
| Help with Finances | Yes = 1 | No = 0 |  |  |  |
| Lost more than 10 lbs in last year | Yes = 1 | No = 0 |  |  |  |
| Self Rating of Health | Poor = 1 | Fair = 0.75 | Good = 0.5 | Good = 0.25 | Excellent = 0 |
| How Health has changed in last year | Worse = 1 | Better/Same = 0 |  |  |  |
| Stayed in Bed at least half the day due to health (in last month) | Yes = 1 | No = 0 |  |  |  |
| Cut down on Usual Activity (in last month) | Yes = 1 | No = 0 |  |  |  |
| Walk outside | <3 days = 1 | ≤ 3 days = 0 |  |  |  |
| Feel Everything is an Effort | Most of time = 1 | Sometime = 0.5 | Rarely = 0 |  |  |
| Feel Depressed | Most of time = 1 | Sometime = 0.5 | Rarely = 0 |  |  |
| Feel Happy | Most of time = 1 | Sometime = 0.5 | Rarely = 0 |  |  |
| Feel Lonely | Most of time = 1 | Sometime = 0.5 | Rarely = 0 |  |  |
| Have Trouble getting going | Most of time = 1 | Sometime = 0.5 | Rarely = 0 |  |  |
| High blood pressure | Yes = 1 | Suspect = 0.5 | No = 0 |  |  |
| CHF / heart attack | Yes = 1 | Suspect = 0.5 | No = 0 |  |  |
| Stroke | Yes = 1 | Suspect = 0.5 | No = 0 |  |  |
| Cancer | Yes = 1 | Suspect = 0.5 | No = 0 |  |  |
| Diabetes | Yes = 1 | Suspect = 0.5 | No = 0 |  |  |
| Arthritis | Yes = 1 | Suspect = 0.5 | No = 0 |  |  |
| Chronic Lung Disease | Yes = 1 | Suspect = 0.5 | No = 0 |  |  |
| BMI | Men  <18.5 and ≥ 30 = 1  25-<30 = 0.5  ≥18.5 -<30 = 0 | Women  <18.5 and ≥ 30 = 1  25-<30 = 0.5  ≥18.5 -<30 = 0 |  |  |  |

CHF: Chronic heart failure. BMI: Body mass index.
