# Supplementary material for: Risk factors of frailty and death or only frailty after intensive care in non-frail elderly patients: a prospective non-interventional study
Source: J Intensive Care. 2019 Oct 30;7:48. doi: 10.1186/s40560-019-0403-3 (PMC6820956; doi:10.1186/s40560-019-0403-3)

**Additional file 2**

**Figures** Details of the variables from the frailty index, reported at admission (remaining non-frail and becoming frail at 6 months) and at 6 months (patients remaining non-frail and becoming frail)

**Figure S1:** basic and instrumental daily living activities.


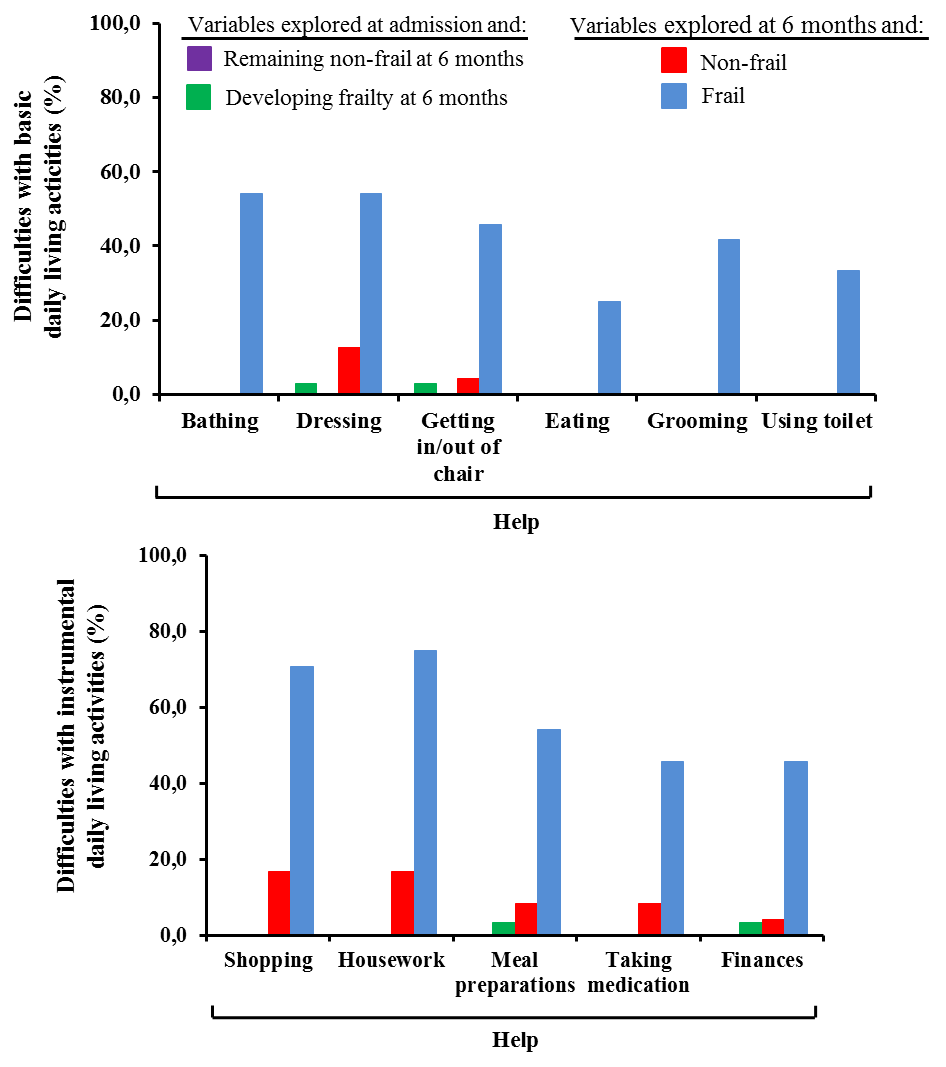


**Figure S2:** Problems related to mobility and strength.


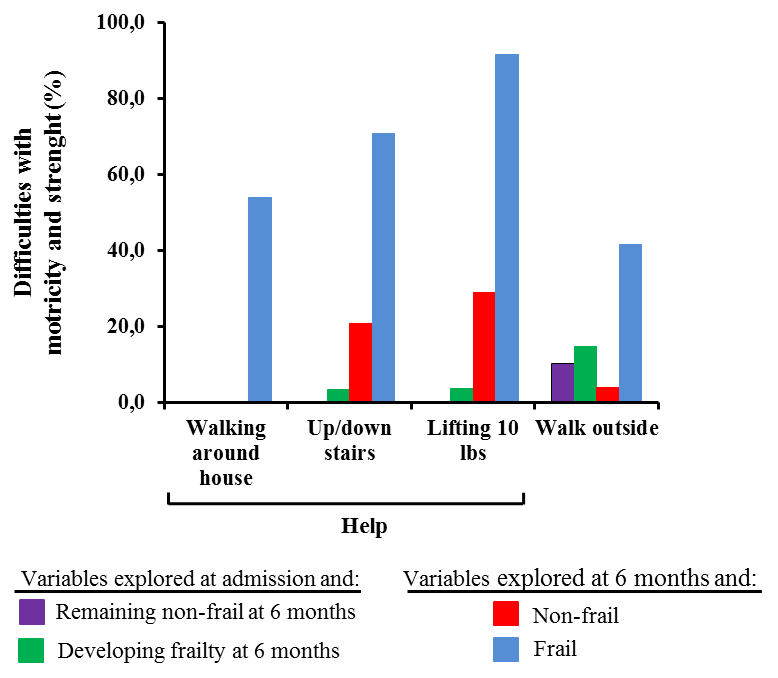


**Figure S3:** Various feeling declared by the patients and trouble getting going.


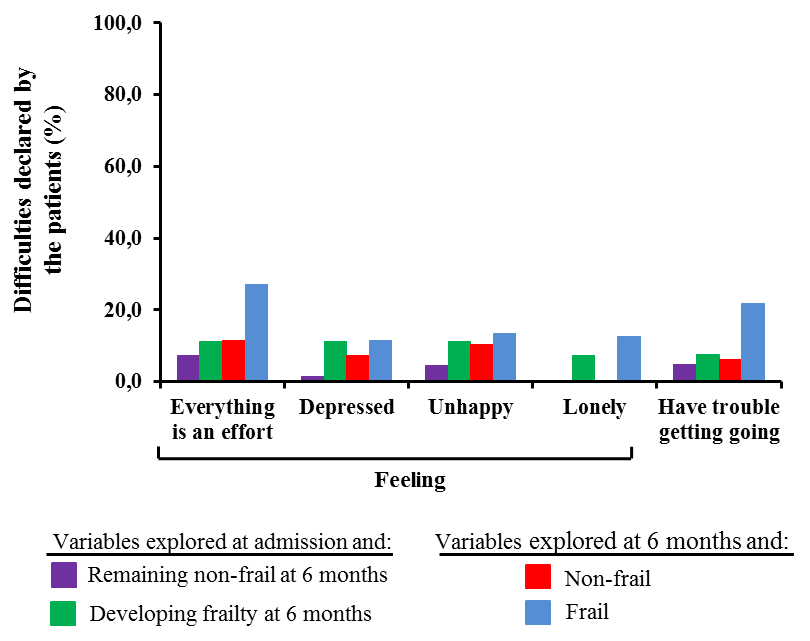


**Figure S4:** Weight loss, altered health status and problems with usual activities health.


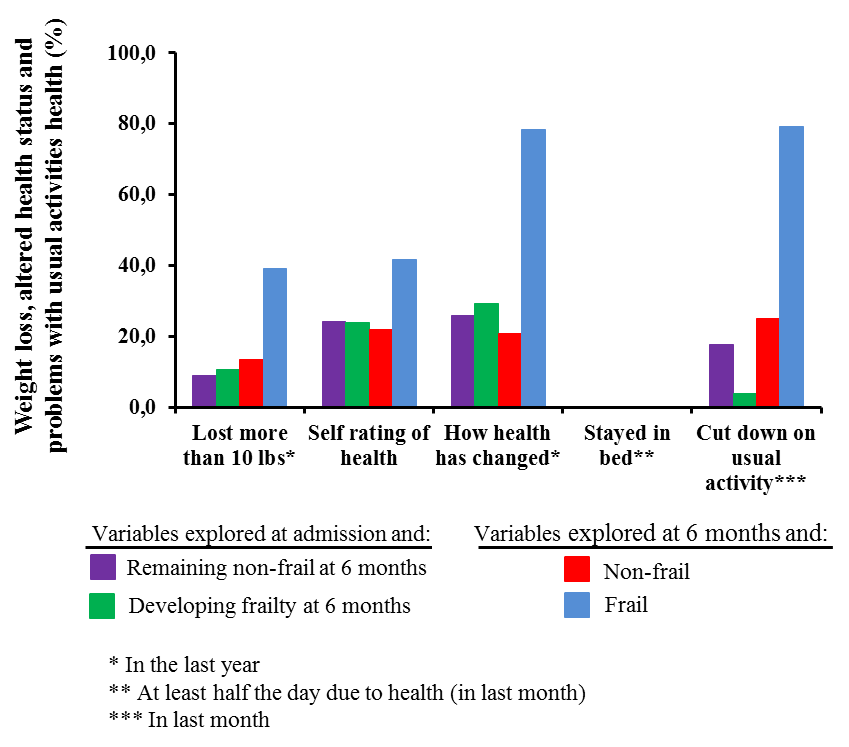


**Figure S5:** Comorbidities.


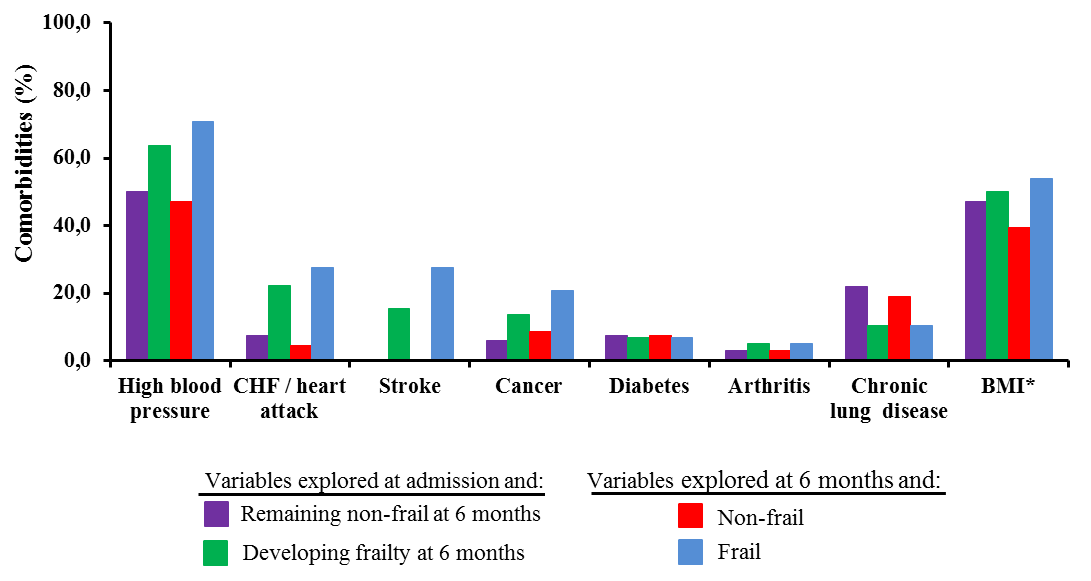

Supplement: Supplementary file 2 — Additional file 2 Details of the variables from the frailty index, reported at admission (remaining non-frail and becoming frail at 6 months) and at 6 months (patients remaining non-frail and becoming frail). Figure S1. Basic and instrumental daily living activities. Figure S2. Problems related to mobility and strength. Figure S3. Various feelings declared by the patients and trouble getting going. Figure S4. Weight loss, altered health status and problems with usual activities. Figure S5. Comorbidities. [file 40560_2019_403_MOESM2_ESM.docx]
